# Analysis of Major Factors Affecting the Quality of Life of the Elderly in Korea in Preparation for a Super-Aged Society

**DOI:** 10.3390/ijerph19159618

**Published:** 2022-08-04

**Authors:** Bo-Ram Kim, Hyang-Hee Hwang

**Affiliations:** 1 Department of Physical Education, Korea University, Seoul 02841, Korea; 2 Department of Sport Science, Kangwon National University, Chuncheon 24341, Korea; 3 Interdisciplinary Program in Biohealth-Machinery Convergence Engineering, Kangwon National University, Chuncheon 24341, Korea

**Keywords:** elderly, health inequality, subjective health status, leisure activities, quality of life

## Abstract

In preparation for the expected super-aged society in 2025, this study attempted to prepare basic data that can help design development measures for the welfare of the elderly so that everyone can prepare for a healthy and happy retirement. Accordingly, the major factors affecting the quality of life of the elderly in Korea were verified. To this end, the questionnaire consisted of 22 questions in total, and a mobile survey was conducted between September and October 2021; in total, 250 copies were used for the final analysis, and the following conclusions are derived. The major factors that were found to determine the quality of life of the elderly were age, subjective health status, monthly household income, leisure activities, and health inequality fairness. It was found that the higher the age, the lower the quality of life. Further, the higher the subjective health status, monthly household income, participation in leisure activities, and perceptions of health inequality as fair, the more the quality of life of the elderly was affected. Therefore, policy support such as leisure activity, health programs, and medical welfare services for the elderly and sufficient attention from our society are all required.

## 1. Introduction

Korea is currently experiencing the fastest population aging in the world as the average life expectancy has increased rapidly. According to Statistics Korea [1], Korea became an aging society in 2017, with the ratio of the elderly aged 65 or older to the total population exceeding 14%, and the elderly population continued to rapidly increase to 7,685,000 in 2019 and 8,125,000 in 2020. As of 2021, the 65-year-old population is about 8.54 million, accounting for 16.5% of the total, and by 2025 it is expected that one in five (20%) of the total population will be over 65; thus, leading to a super-aged society. However, in Korea, unlike other developed countries, the social security system—such as that providing for housing, care, and basic livelihood security—has not been systematically laid out, even in the current situation where the elderly population is rapidly increasing.

This situation greatly affects the lives of the elderly, and it has led to the emergence of various social problems, such as an increase in family support costs, an increase in elderly poverty rates, elder abuse and loneliness, and an increase in suicidal thoughts and ideation among the elderly [2].

### 1.1. Prior Research Related to Quality of Life

According to role theory, people perceive themselves as social beings and develop a self-concept while performing roles [3]. However, old age comes with difficulties caused by role loss due to various environmental changes such as retirement and separation from children [4]. It is also accompanied by issues with health problems and the functions of various body organs due to alienation, depression, and the deterioration associated with aging rapidly.

Recently, there have been various movements aiming to improve the quality of life of the elderly by addressing their physical, psychological, and social problems [5]. Quality of Life (QOL) starts with philosophical discourse. It began to be measured in the 1980s as a result of intervention outcomes, with a particular focus on the fields of psychology, physical education, medicine, and social welfare [6]. The World Health Organization (WHO) defines quality of life as a perception of what is relevant to one’s goals, expectations, standards, and interests in the context of the culture and value system in which one lives [7]. QOLis also explained as a broad concept that encompasses an individual’s physical and psychological health, independence, social relationships, and personal belief environment [8]. QOL also refers to subjective well-being that includes concepts such as happiness, life satisfaction, and positive emotions. It can be said to be an index that comprehensively explains these concepts.

Ultimately, QOL can be said to be a very important concept in grasping an individual’s life success because it has a direct impact on the achievement of one’s life goals [9]. In addition, subjective evaluations are more important than objective evaluations when assessing an individual’s QOL [10]. In Korean society, starting with the aging of the population, with the current well-being trend, measuring the happiness and QOL of the elderly through objective factors alone has limitations. As subjective views on QOL begin to be emphasized, research is moving toward multifaceted investigations [11,12].

Meanwhile, multidimensional factors such as health and individual values must also be considered as factors related to the QOL of the elderly. They are viewed from an ecological perspective, including environmental opportunities due to demographic, physical, and psychological factors as well as personality and intellectual ability. In particular, in terms of physical characteristics, it has been reported that the subjective health status of an elderly person has an important influence on their QOL [4,13]. Subjective health status refers to an individual’s perception of their overall health status. Research has shown that the more the elderly themselves positively perceive their levels of health, the higher their QOL [14]. Recently, studies have shown that an individual’s subjective health level has higher reliability for indicating one’s cognitive health level than objective evaluation, leading to greater weight being placed on one’s subjective health status [15]. According to a study by Choi and Lee [16], the QOL of the elderly is higher when their subjective health status is positive (i.e., when they believe that they are healthy). A study by Jung, Lee, and Sin [17] has also shown that the subjective health status of the elderly is a major variable affecting their QOL. This emphasizes that elderly people should manage their own health so that they can lead healthy lives after retirement. Systematic health education programs should be provided through various institutions in the community. Being healthy is a crucial part of improving the QOL of the elderly. Enjoying the best health conditions possible is a basic right for all humans regardless of race, region, socioeconomic level, or political beliefs [18].

Looking at previous studies performed in neighboring countries on major factors affecting the QOL of the elderly, China has made progress in targeting air pollution and health costs [19]. It has been reported that regional differences in the old-age dependency ratio, age, family size, self-assessment health status, and income due to an aging population are factors influencing medical expenses [20]. In addition, as non-communicable disease multimorbidity emerged as a medical problem for the Indian people, they argued for health equity [21,22,23]. Previous studies revealed that in most countries of Asia, out-of-pocket expenses did not protect the poor. Health inequality problems occur depending on the household standard of living and regional heterogeneity, thus affecting QOL.

Our society is experiencing health inequality problems according to income level and region of residence. Health inequality refers to inequality in life options that can lead to a difference in health according to socioeconomic status, which can lead to the polarization of health outcomes [24]. Evans et al. [25] have pointed out that the health gap according to socioeconomic conditions is a sensitive indicator of the level of inequality or fairness in society as a whole. Health not only means the absence of disease but also reflects society and the environment. If one’s basic right to health is not guaranteed, it will have a negative impact on individual happiness and quality of life, thus negatively affecting the development of local communities and national competitiveness [26]. Furthermore, the inequality of opportunity decreases life satisfaction [27]. Since health is a very important influencing factor in improving the QOL of the elderly, it is necessary to predict the relationship between the level of perception of health inequality (seriousness and fairness) and QOL and make efforts to respond to health inequality.

Meanwhile, in recent years, there have been various movements to improve the QOL of elderly people through participation in leisure activities to solve physical, psychological, and social problems [4]. In general, “leisure time” means free time excluding physiologically essential periods from the remaining hours after daily work [28]. For the elderly who do not work, leisure time becomes “all of life” rather than the time left after work [29]. Therefore, how elderly people use their leisure time is particularly important as they have a sudden increase in such time after retirement. It is known that participation in leisure activities during old age has a physiologically desirable effect, thus improving QOL [30].

Participation in leisure activities has a positive effect on the health of the elderly. Active and productive leisure activities can improve the QOL, including physical and mental health [31,32]. In addition, participation in leisure activities can enhance the elderly’s ability to smoothly participate in social roles, thus improving their QOL [33]. In other words, active participation in leisure activities can help solve the difficulties caused by the loss of roles due to various changes in old age [5,34]. A study by Cho and Hur [35] showed that the QOL related to health was greatly improved for the elderly who participated in regular physical leisure activities, while the QOL of the elderly who did not participate in leisure activities was decreased. As such, many previous studies have reported that participation in leisure activities has a positive effect on the QOL of the elderly [11,36,37,38]. In addition, demographic characteristics (gender, age, income level, academic background, etc.) and environmental characteristics can lead to differences in happiness and QOL [39,40]. Demographic characteristics and variables have also been studied as predictors of QOL [41,42,43]. Based on previous studies, we intend to identify determinants and predictors of the QOL in the elderly in detail.

### 1.2. Purpose and Expected Effect of the Study

At this point, we should look at the old age phenomenon as a social problem rather than an individual problem. In the future, it will be necessary to look at the lives of future old age that everyone will face more realistically and devise various ways to improve the QOL of people at such an age.

Korea is set to become a super-aged society in 2025. At present, the perspective on old age is rapidly changing from extending life to an interest in healthier old age life and quality of life. Accordingly, research on the quality of life of the elderly is actively being conducted, but as the quality of life of the elderly is also changing in line with the rapidly changing trend of the times, related research should be conducted continuously as well. The quality of life in old age has now become a key paradigm in our society, and it is very important to find out the main factors affecting the quality of life, especially as a super-aged society is about three years away. Therefore, the purpose of this study is to examine the main factors that have recently been shown to affect the QOL of the elderly in relation to health and leisure activities. To this end, demographic characteristics variables, fairness and seriousness of health inequality, subjective health status, and leisure activities are set as major variables, and the ranking of the major determinants is to be investigated. This study is expected to serve as a basis for interventions to predict the major factors affecting the quality of life of the elderly in preparation for a super-aged society and to improve the quality of life of the elderly. It is also intended to serve as basic data to supplement the limitations of existing research and help design development plans for the welfare of the elderly who can prepare for a healthy and happy old age.

Based on this, the research questions are established as follows: First, how do socio-demographic characteristics and variables of Model 1 affect the QOL of the elderly? Second, how does the effect on the QOL of the elderly change when the degree of health inequality (severity, fairness) of Model 2 is additionally added? Third, how does the effect on the QOL of the elderly change when subjective health perception and leisure activities of Model 3 are added?

Globally, the aging of the elderly is a big issue. Thus, research dealing with their QOL is very important. In particular, considering that Korean society is aging rapidly compared to other countries, it is meaningful to derive relative major factors affecting the quality of life of the elderly. Moreover, variables have been set in consideration of the characteristics of the elderly in Korea who have difficulties related to economic poverty, health inequality, and health status. These research attempts will help us establish and seek effective welfare policies to improve the happiness and QOL of the elderly in the future. This study is expected to serve as a stepping stone for preparing and welcoming the ultra-aging era.

## 2. Materials and Methods

### 2.1. Participants and Sampling

For the ethical considerations of this study, the research was carried out after receiving the final approval for research ethics from the Ethics Committee in August 2021. First, the comprehension, appropriateness, and expected time required for the questionnaire questions were reviewed for 100 senior citizens aged 65 and over, mainly at local health clinics and village halls, and the content validity of the measurement questions was checked next. After that, the questionnaire was finally composed by modifying and supplementing the measurement items. The main survey was commissioned by Rende Research (www.rende.co.kr, accessed on 12 July 2021), a specialized research institute, and a mobile survey was conducted for two months in September and October 2021. In total, 300 copies were distributed to the elderly aged 65 or older, and the results of the socio-demographic characteristics of 250 people used in the actual analysis are presented in Table 1.

### 2.2. Validity and Reliability of the Measurement Tool

Questionnaires were used as measurement tools to achieve the purpose of this study, and the suitability and content validity of the questions were verified through expert meetings with three physical education professors and two leisure doctors to determine the suitability and validity of the questions. The questionnaire consisted of socio-demographic characteristics variables (gender, age, educational background, income, household type), health inequality severity, health inequality fairness, subjective health status, leisure activities, and quality of life scale.

Specifically, to measure health inequality severity and health inequality fairness, we used the subjective health inequality perception measurement tool developed by Kim [44]. The health inequality severity is a four-item single factor, and an example item is “How serious do you think health inequality between men and women is in our society?” Health inequality fairness consisted of one item, and the content of the item consisted of “Do you think the difference in health level between social classes and regions in our society is fair?” The response form for each question consisted of a 5-point Likert scale (1 point, not at all ~5-point, very much so).

For the subjective health status scale, we used a questionnaire described in a study by Lee [45] that consists of one item: “What is your current subjective health status?” The response form was composed of a 5-point Likert scale (1 point, not at all ~5-point, very much so). In addition, the question about participation in leisure activities consisted of one item: “Do you currently engage in leisure activities?” and the response form was composed of ‘yes’ and ‘no’. For the QOL scale, we used a questionnaire from the study by Ko [46], which consists of ten items in total among two sub-factors: five items for life satisfaction and five items for life expectation. Examples of items include “I am very satisfied with my life” and “My life will be closer to my ideals in the future”. The response form was composed of a 5-point Likert scale (1 point, not at all ~5-point, very much so). The compositions of the specific questionnaire items are shown in Table 2.

Next, confirmatory factor analysis was performed to verify the construct validity between the latent variables and measurements. For the absolute fit indices used to evaluate the fit, it was checked whether the model was suitable for the data while centering on the Chi-square, RMSEA, GFI, SRMR indices, the incremental fit indices NFI, CFI, and the TLI indices. As a result of the analysis, as presented in Table 3, the fitness index was χ^2^ = 133.620, *df* = 34, GFI = 0.948, NFI = 0.941, CFI = 0.955, TLI = 0.941, RMSEA = 0.076, SRMR = 0.042, which met the standard. In addition, Cronbach’s α was calculated to verify the reliability of the measurement tool. Cronbach’s α of health inequality severity was 0.788, and Quality of life was 0.854, so reliability was secured.

### 2.3. Data Processing Method

The SPSS 25.0 (IBM Corporation, New York, NY, USA) and AMOS 25.0 programs (IBM Corporation, New York, NY, USA) were used to analyze the major factors affecting the QOL of the elderly. The specific analysis method is as follows. First, a frequency analysis was performed to determine the general characteristics of the subjects. Second, to verify the dimensionality and validity of the factor structure of variables, confirmatory factor analysis was performed using the AMOS 25.0 program. In addition, Cronbach’s α coefficient was calculated to assess reliability. Third, descriptive statistical analysis was performed to find the mean, standard deviation, skewness, and kurtosis of variables. Fourth, Pearson’s correlation analysis was performed to identify correlations between variables. The problem of multicollinearity was also assessed. Finally, hierarchical regression analysis was performed to find out the major factors affecting the QOL. Variables composed of nominal scales were changed to dummy variables and then analyzed. The significance level for all statistical analyses was set at *p* < 0.05.

## 3. Result

### 3.1. Descriptive Statistical Analysis

Descriptive statistical analysis was performed to examine the minimum, maximum, mean, standard deviation, skewness, and kurtosis of variables used in the study. As a result of the analysis (Table 4), the absolute value of skewness did not exceed 3, and that of kurtosis did not exceed 8, satisfying the criteria of Kline [47]. Thus, a normal distribution can be assumed, Table 4.

### 3.2. Correlation

The purpose of this study is to analyze the main factors affecting the quality of life of the elderly in Korea in preparation for a super-aged society. To this end, a correlation analysis between variables was first conducted. As a result of the analysis, it was found to be partially significant, and it showed a positive (+) and negative (−) correlation within the significance level. It was also confirmed that there was no problem in multicollinearity as the correlation coefficient value did not show a high correlation of 0.8 or more, Table 5.

### 3.3. Variables Affecting Quality of Life in the Elderly

Table 6 lists the results of the hierarchical regression analysis conducted to determine the effect on the quality of life by subdividing it step by step. The first stage was introduced and analyzed in the order of socio-demographic variables (Model 1), the second stage was the health inequality variable (Model 2), and the third stage was the subjective health status and leisure activities (Model 3). Statistically significant results were found in Model 1 (*F =* 30.919, *p* < 0.001), Model 2 (*F* = 23.669, *p* < 0.001), and Model 3 (*F* = 24.678, *p* < 0.001). Therefore, the regression equation was confirmed to be statistically significant. As shown in Model 1, Model 2, and Model 3, at least one of the variables input as an independent variable has a significant effect on the dependent variable.

First, looking at Model 1, including demographic variables, *R^2^* was 0.388, which had an explanatory power of 38.8%. The *F* value was 30.919 (*p* < 0.001), showing a statistically significant effect on QOL. Specifically, age (*t* = −8.736, *p* < 0.001) was found to have a statistically significant negative (−) effect on QOL. Monthly household income (*t* = 6.030, *p* < 0.001) was found to have a positive (+) effect on the quality of life. On the other hand, it was confirmed that gender, educational level, and household type among demographic variables did not significantly affect the QOL.

Next, Model 2 was analyzed by adding health inequality severity and health inequality fairness as variables related to health inequality in Model 1. As a result, the *R*^2^ was 0.406, which showed an explanatory power of 40.6%. The amount of change in *R*^2^ increased by 1.8% compared to Model 1, which was significant (Δ*R*^2^ under the statistical significance level). In addition, the amount of change in *F* was 3.781 (*p* < 0.001), indicating that the variables added to Model 2 had a significant effect on the QOL. Specifically, as in Model 1, age (*t* = −8.095, *p* < 0.001) had a negative (−) effect on the QOL, and monthly household income (*t* = 5.910, *p* < 0.001) had a positive (+) effect. In addition, among additional input variables, health inequality fairness (*t* = 2.625, *p* < 0.01) was found to have a positive (+) effect on the QOL. It was confirmed that health inequality severity did not have a significant effect.

Finally, Model 3 was analyzed by additionally adding subjective health status and leisure activities. As a result, *R*^2^ was 0.481, which showed an explanatory power of 48.1%. The amount of change in *R*^2^ increased by 7.5% compared to Model 2, which was significant (Δ*R*^2^ under the statistical significance level). In addition, the amount of change in *F* was 17.153 (*p* < 0.001), indicating that variables added to Model 3 had a significant effect on the QOL. Specifically, as in Model 2, age (*t* = −7.565, *p* < 0.001) had a negative (−) effect on the QOL. Monthly household income (*t* = 3.049, *p* < 0.01) and health inequality fairness (*t* = 3.034, *p* < 0.01) were found to have a positive (+) effect on the QOL. In addition, among additional input variables, subjective health status (*t* = 4.798, *p* < 0.001) and leisure activities (*t* = 3.088, *p* < 0.01) were found to have positive (+) effects on the QOL.

We now summarize the relative influence on the quality of life of the elderly in Korea: Age (*β* *=* −0.391, *p* < 0.001) was found to have the greatest influence on quality of life, followed in descending order by subjective health status (*β* *=* 0.261, *p* < 0.001), monthly household income (*β* *=* 0.174, *p* < 0.01), leisure activities (*β* *=* 0.156, *p* < 0.01), and health inequality fairness (*β* *=* 0.150, *p* < 0.01). In other words, the main factor that degrades the quality of life of the elderly is age, and the factor that has the greatest influence on improving the quality of life was found to be subjective health. In addition, the Dublin–Watson was 1.660, which was judged to be more suitable as it was closer to 2, thus confirming that there was no correlation between the residuals. The tolerance limit (Tolerance) was 0.665~0.965, which was found to be a suitable standard for a value of 0.1 or higher, and the variance expansion index (VIF) was in the range of 1.032~1.504; as it was less than 10, it was confirmed that there is no problem with multicollinearity.

## 4. Discussion

The purpose of this study is to identify the main factors affecting the quality of life among the elderly in Korea in preparation for a super-aged society, which the nation is projected to become by 2025. To this end, the first stage considered demographic variables, the second stage considered health inequality-related variables, and the third stage examined how the influence changes at each stage by examining the influence of subjective health status and leisure activities. As a result, it was found that the quality of life of the elderly was affected in the order of age, subjective health status, monthly household income, leisure activities, and health inequality fairness. Specifically, when discussed according to the step-by-step analysis, in Model 1, age had a negative effect on the quality of life, whereas monthly household income had a positive effect. These results are the same as those reported in previous studies that higher age is associated with lower quality of life [17,48,49]. A study by Kim and Jeong [50] reported that an increase in the age of the elderly degrades the quality of life and that it is associated with more instances of not receiving social support in various areas, thereby reducing life satisfaction. The elderly face decreased overall quality of life due to mobility difficulties and physical weakness [51], and physical, mental, psychological, and social changes caused by aging can threaten an individual’s emotional state [52]. Therefore, since aging anxiety caused by an increase in age is a cause of further deteriorating the quality of life [53], there is a need for efforts to improve quality of life by reducing aging anxiety.

In addition, a study by Jung et al. [17] showed that the higher the household income of the elderly, the higher their quality of life. Even today, when economic gains have been made in general compared to the past, the fact that income still affects the quality of life has great implications for the elderly’s economic poverty. The research results of Zhang et al. [54] also showed that the higher the income level, the higher the quality of life. A study by Hawro et al. [55] showed that per capita household income had a significant correlation with all areas of quality of life, including psychological and social relationships, and the lower the income, the lower the quality of life. Diener [56] mentioned that there is a positive relationship between income and subjective well-being, quality of life, and happiness. As such, when the income of the elderly is high, their quality of life and level of happiness are high as well [4,57]. People with low incomes are more likely to experience stress and frustration due to dissatisfaction with social comparison [58]. However, after economic income increases above a certain level, the quality of life and happiness of the elderly no longer improve [59]. In other words, rather than the meaning of “unconditionally, the higher the income, the higher the quality of life”, it should be interpreted as a factor that positively contributes to the quality of life of the elderly at a satisfactory level of income that will not feel the pressures of poverty. Therefore, welfare policies to further expand employment opportunities for the elderly, raise basic pensions and benefits, and provide economic support should be discussed from various angles.

Next, in Model 2, age and monthly household income were affected as in Model 1, and it was also found that health inequality fairness had a positive effect on the quality of life. That is, the fairer one perceives the difference in health levels between classes and regions in our society to be, the higher the quality of life is. The health gap according to socioeconomic conditions causes social inequality, and if the people’s basic rights to health are not guaranteed, this will negatively affect their quality of life [25]. Social inequality intensifies health inequality, and various inequalities degrade individual life satisfaction and quality of life [60,61]. Namely, the fairer the level of health between classes and regions without inequality, the better the quality of life will be. Lim, Ku, and Choi [62] emphasized that policy efforts to resolve inequality can lead to increased happiness levels throughout society. In this respect, Korean society has room to reconsider whether all people in our society are equally enjoying the various benefits of a healthy life. This is because people in regions with relatively low education and income levels and high deprivation complain of health inequality [44]. Health inequality is not a natural phenomenon but the result of a combination of poor social policies and programs and unfair economic systems, and it is both caused by and reinforces socioeconomic inequality [63]. Therefore, active social attention is required to improve the quality of life of Korean people, including the elderly.

Finally, in Model 3, it was found that both the additionally-input subjective health status and leisure activities had a significant effect on the quality of life. Therefore, the main factors influencing the quality of life were found to be, in descending order: age, subjective health status, monthly household income, leisure activities, and health inequality fairness. A study by Jung et al. [17] showed that the factor with the greatest influence on the quality of life of the elderly was subjective health status. In addition, in a study by An and Choi [64], subjective health status was continuously derived as an important factor throughout 2007, 2010, and 2014. In this study as well, subjective health status was found to be a major factor in improving the quality of life, thus suggesting that the health of the elderly still importantly contributes to improving the quality of life. In general, it is reported that the better the health of the elderly, such as fewer chronic diseases, subjective health status, and ability to perform daily activities, the higher their quality of life and feelings of happiness [4,39,45,65,66]. The healthier the elderly, the more positive feeling they will have about their lives and the more they tend to be satisfied with their lives, and their quality of life will be higher [67]. As such, the more positively the elderly perceive their health status, the higher their quality of life, so it is very important to maintain good health. Therefore, elderly people in poor health need medical benefits and policy support for health promotion [53]. Further, people should voluntarily pay attention to their health and try to lead a healthy life through regular exercise.

Kim and Lim [68] showed that the quality of life changed positively through participation in various leisure activities. It has also been reported that the older the elderly who participate in active leisure activities, the higher the gains in the quality of life [69,70]. Regular leisure activities play an important role in improving the health-related quality of life of the elderly [35] and contribute very positively to improving the quality of life through the formation of social relationships and an active life [57]. According to the Activity Theory, people seek to find new roles that can replace the roles they have lost in old age, and active social activities or leisure activities improve the life satisfaction of the elderly [71,72]. Therefore, participation in leisure activities should be actively recommended as a very positive activity that can replace the loss of roles in old age.

In summary, it was found that age is the main factor that reduces the quality of life of the elderly, while the factor with the largest influence on improving quality of life is subjective health status. Aging is a natural process, and everyone faces old age. Therefore, support from all of us and various government programs will be needed to support reducing the worries about aging and help people form positive values. In addition, health education programs should be actively implemented so that the elderly can form positive health perceptions. It is very important to promote a healthy life without health inequality through participation in various leisure activities at present when the projected super-aged society in 2025 is not far away. To this end, equal resource allocation of systematic medical and welfare services is required for the elderly. If our society can become interested enough in this important issue that will eventually affect us all, the quality of life of the elderly can be improved even more. Finally, the government’s benefits for financial stability and health promotion of the elderly should be guaranteed to anyone. The vision and direction of these welfare policies should be placed on improving the QOL of people. In this respect, the results of this study are thought to be helpful in seeking customized welfare benefits and directions for the policy-establishment process to improve the QOL of the elderly. In addition, as income, fairness, health, and leisure activities are important factors affecting the QOL of the elderly, the results of this study could be used as basic data for formulating effective policies that reflect the satisfaction and needs of the elderly in Korea.

## 5. Conclusions

This study attempted to contribute basic data to help design development measures at the welfare level of the elderly so that everyone can prepare for a healthy and happy retirement in preparation for a super-aged society in 2025. Accordingly, the following conclusions were drawn as a result of confirming the main factors affecting the quality of life for the elderly in Korea. The main factors determining the quality of life of the elderly were found to be in descending order: age, subjective health status, monthly household income, leisure activities, and health inequality fairness. It was found that the higher the age, the lower the quality of life; further, the higher the subjective health status, monthly household income, participation in leisure activities, and perceptions of health inequality as fair, the more it affected the quality of life of the elderly. Therefore, generous support such as customized leisure education services and health programs for the elderly is required in terms of efforts and policies for individual health management. Social and policy interests and efforts should complement such individual efforts.

This study is meaningful in that it derives the relative major factors affecting the quality of life of the elderly in preparation for the upcoming super-aged society. In addition, the validity and reliability of the survey tool were secured. However, it has a limitation in that it has not secured more sampling due to the COVID-19 situation. In addition, for elderly people, it may be difficult to answer the questionnaire when the number of questions increases, so there is a limit to constructing and measuring various variables.

To this end, I would care to make the following suggestions for future research. In the follow-up study, a larger number of samples should be secured for the elderly, and the characteristics of the elderly generation should be specifically identified through comparative studies between generations such as the middle-aged and the prospective elderly. It will also be necessary to verify the empirical effect by developing various content and programs to improve the quality of life.

## Figures and Tables

**Table 1:** Demographic characteristics.

Variable	Category	*n*	%	Variable	Category	*n*	%
Gender	Male	109	43.6	Age	67 ± 1.40	250	100.0
	Female	141	56.4				
Educational background	Middle school graduate or lower	72	28.8	Monthly household income (won)	Below 2 million	36	14.4
					2–2.99 million	43	17.2
	High school graduate	114	45.6				
					3–3.99 million	76	30.4
	Junior college or higher	64	25.6				
					4–4.99 million	64	25.6
					5 million or above	31	12.4
Household type	Live alone	34	13.6	Participation in leisureactivities	Yes	140	56.0
					No	110	44.0
	Live together	216	86.4				
Total		250	100.0	Total		250	100.0

**Table 2:** Survey item compositions.

Category (Number of Questions)		Example of Questions
Socio-demographic characteristics		Gender, age, educational background, monthly household income, and household type
Health inequality severity (4)		How serious do you think health inequality between men and women is in our society? How serious do you think health inequality is between poor and rich people in our society? How serious do you think health inequality is between people with low and high levels of education in our society? How serious do you think health inequality is between city dwellers and rural residents in our society?
Health inequality fairness (1)		Do you think the difference in health level between social classes and regions in our society is fair?
Subjective health status (1)		What is your current subjective health status?
Leisure activity (1)		Do you currently engage in leisure activities (watching and participating in culture and arts, watching and participating in sports, tourism, and travel)?
Quality of life (10)	Life satisfaction (5)	I am very satisfied with my life. The conditions of my life are very good. All in all, my life is close to my ideal. So far, I have achieved the important things I want in life. If I were to be reborn, I would keep my life pretty much the same.
	Life expectation (5)	My life will be closer to my ideals in the future. The conditions of my life will be better in the future. I will be more satisfied with my life in the future. In the future, I will achieve more important things that I want in my life. In the future I will develop and grow.

**Table 3:** Fit of the measurement tool.

Variables	GFI	NFI	CFI	TLI	RMSEA	SRMR
Standard	≧0.90	≧0.90	≧0.90	≧0.90	≦0.10	≦0.08
Model fit	0.948	0.941	0.955	0.941	0.076	0.042
	χ^2^ = 133.620 *(df* = 34, *p* = 000)					

**Table 4:** Descriptive Statistics of Variables.

Variables	Min	Max	Mean	SD	Skewness	Kurtosis
Gender	1.00	2.00	1.56	±0.50	−0.26	−1.94
Age	65	69	67.01	±1.40	0.04	−1.30
Education	1.00	3.00	1.97	±0.74	0.05	−1.16
Income	1.00	5.00	3.04	±1.23	−0.15	−0.88
House type	1.00	2.00	1.84	±0.37	−2.13	2.58
Health inequality severity	1.60	5.00	3.49	±0.70	−0.28	−0.09
Health inequality fairness	1.00	5.00	3.49	±0.88	−0.34	0.12
Subjective health status	1.00	3.00	2.04	±0.60	−0.02	−0.23
Leisure activity	1.00	2.00	1.44	±0.49	0.24	−1.96
Quality of life	1.00	4.80	3.40	±0.78	−1.19	0.76

**Table 5:** Correlation analysis

Variable	1	2	3	4	5	6	7	8	9	10
gender	1									
age	0.030	1								
education	−0.303 **	−0.234 **	1							
income	−0.119	−0.169 **	0.333 **	1						
household type	−0.090	−0.063	0.058	0.169 **	1					
inequality severity	−0.067	−0.195 **	0.026	0.032	0.012	1				
inequality fairness	−0.044	−0.268 **	0.126 *	0.141 *	0.128 *	0.201 **	1			
health status	−0.110	−0.086	0.215 **	0.491 **	−0.029	−0.037	0.020	1		
leisure activity	−0.005	−0.287 **	0.037	0.131 *	0.119	0.268 **	0.111	0.084	1	
quality of life	−0.051	−0.520 **	0.251 **	0.421 **	0.131 *	0.060	0.293 **	0.400 **	0.313 **	1

* *p* < 0.05, ** *p* < 0.01.

**Table 6:** Hierarchical Regression Analysis on Quality of Life.

	Variable	B	SE	*β*	*t*	*F*	Δ*F*	*R* ^2^	Δ*R*^2^
Model 1	(constant)	19.359	2.038		9.500	30.919 ***	30.919 ***	0.388	0.388
	gender	0.028	0.084	0.018	0.338				
	age	−0.255	0.029	−0.453	−8.736 ***				
	education	0.035	0.051	0.039	0.690				
	income	0.195	0.032	0.326	6.030 ***				
	household type	0.108	0.118	0.047	0.922				
Model 2	(constant)	18.517	2.165		8.554	23.669 ***	3.781 ***	0.406	0.018
	gender	0.023	0.083	0.014	0.272				
	age	−0.244	0.030	−0.433	−8.095 ***				
	education	0.028	0.051	0.031	0.550				
	income	0.190	0.032	0.317	5.910 ***				
	household type	0.076	0.117	0.033	0.654				
	health inequality severity	−0.072	0.058	−0.063	−1.228				
	health inequality fairness	0.124	0.047	0.138	2.625 **				
Model 3	(constant)	16.056	2.109		7.615	24.678 ***	17.153 ***	0.481	0.075
	gender	0.040	0.078	0.025	0.510				
	age	−0.220	0.029	−0.391	−7.565 ***				
	education	0.025	0.048	0.028	0.528				
	income	0.104	0.034	0.174	3.049 **				
	household type	0.112	0.111	0.049	1.010				
	health inequality severity	−0.096	0.056	−0.084	−1.694				
	health inequality fairness	0.135	0.045	0.150	3.034 **				
	subjective health status	0.342	0.071	0.261	4.798 ***				
	leisure activity	0.129	0.042	0.156	3.088 **				

Durbin-Watson = 1.660, ** *p* < 0.01, *** *p* < 0.001.

## Data Availability

Not applicable.
